# Fibrin, and Its Relation to the Blood and the Tissues

**Published:** 1870-01

**Authors:** 


### ARTICLE IV.

### *Fibrin, and its relation to the Blood and the Tissues.*

At a meeting of the "Baltimore Medical Association" Prof. Henry R. Noel of the Baltimore College of Dental Surgery, gave the following views in regard to fibrin in the blood, the subject under discussion being the "Causes of Sudden Death."

I. That fibrin is the "formed material" of the "germinal matter" of Dr. L. S. Beale, and is, therefore, to be placed in the histogenetic, rather than the histolytic class.

II. That when found in the blood, it is to be considered as a product of the white blood corpuscle, except in case of disease, when it may be found in the tissue, and absorbed into the blood vessels.

III. That whether found in the blood current, or in tissues, and absorbed into the blood current, it is in each and every instance an *abortion*, and marks a degree of lowered vitality of the germinal matter of the tissue, or of the blood. The "germinal matter" in the blood, i. e. the white blood corpuscles, fails to develope normally into the red corpuscles, but *aborts*, and gives fibrin as the product of this abortion.

IV. That the germinal matter of the tissues, and especially the connective tissue corpuscles of Virchow, (which are only masses of germinal matter,) often exhibit a like abortive effect at development, and that in this instance also, the fibrin arises as the product of the abortion.

V. That there is no such thing as a fibrinous exudation from the blood into the tissues, but the fibrin may be, and often is, found in the tissues, and then absorbed into the blood.

VI. That whether found in blood or tissue primarily, it must sooner or later be oxidised and carried off as urea, carbonic acid, &c.; that being a product of abortive histogenesis, it could subserve no useful purpose in the economy save the chemical one of producing heat.

VII. That in inflammation, such as the phlegmons, pneumonias, &c., the fibrin of the buffy coat was formed principally in the tissues diseased, and not in the blood; that being formed locally in the tissue diseased, it was by osmotic action taken by the lymphatics in the blood current.

VIII. That being taken into the blood, it is, in reality, a foreign element, and must be at once disposed of; that this can only be done by oxidation, and the rapid oxidation of the fibrin is one source of the heat in the fevers which mark these phlegmasiæ.

IX. That in the inflammatory process the first step is the local formation of fibrin; the second is its absorption by the lymphatics; the third is its oxidation in the blood; the fourth is its elimination by the urinary organs as urea, uric acid, &c.; that the increased respiration and increased circulation are necessary to supply the increased demand for oxygen; the heat is the necessary consequence of the increased oxidation.

X. That as long as the local production can be removed by the local lymphatics, and this fibrin oxidised in the blood and excreted by the kidney, there will be no local accumulation; but should there be deficient oxidation, or deficient action of lymphatics, there will be a local accumulation at the point of formation, and this fibrin then and there coagulating will give us the hepatization of pneumonia and phlegmons.

XI. That fibrin in the blood cannot, therefore, be considered a normal or physiological element of the blood, but an abnormal or pathological one; that its presence in blood or tissue tells a history of arrested development, with abortive histogenesis; that the amount present is the accurate measure of pathological change, or the degree of aberration from the true physiological condition.

*Physiological blood should have no fibrin.*

XII. That, therefore, before we investigate embolism as a cause of sudden death, it would be but just to investigate the action of those causes which devitalise the germinal matter ot blood and tissue, and lead to the production of that abnormal element, fibrin, the coagulation of which in the heart, Dr. Richardson thinks, is so frequent a cause of death. Coagula are found in the heart after death, from nearly every cause, save lightning; and it was a mooted question whether the fibrin was not formed *during dissolution*, and after *somatic*, but *before molecular death.* The clot, in blood drawn from the body, shows the fibrin and white corpuscles inversely proportional, and is not, therefore the fibrin formed from these, and if so, may it not be formed during and after the drawing of the blood, and be in fact the result of the changed and abnormal relations of blood, vessels, and atmosphere?

